# An Investigation of Insider Threat Mitigation Based on EEG Signal Classification

**DOI:** 10.3390/s20216365

**Published:** 2020-11-08

**Authors:** Jung Hwan Kim, Chul Min Kim, Man-Sung Yim

**Affiliations:** Korea Advanced Institute of Science and Technology, Department of Nuclear and Quantum Engineering, Daejeon 34141, Korea; poxc@kaist.ac.kr (J.H.K.); usekim00@kaist.ac.kr (C.M.K.)

**Keywords:** nuclear security, insider threat, electroencephalography, machine learning, implicit intention, subject-wise classification

## Abstract

This study proposes a scheme to identify insider threats in nuclear facilities through the detection of malicious intentions of potential insiders using subject-wise classification. Based on electroencephalography (EEG) signals, a classification model was developed to identify whether a subject has a malicious intention under scenarios of being forced to become an insider threat. The model also distinguishes insider threat scenarios from everyday conflict scenarios. To support model development, 21-channel EEG signals were measured on 25 healthy subjects, and sets of features were extracted from the time, time–frequency, frequency and nonlinear domains. To select the best use of the available features, automatic selection was performed by random-forest-based algorithms. The k-nearest neighbor, support vector machine with radial kernel, naïve Bayes, and multilayer perceptron algorithms were applied for the classification. By using EEG signals obtained while contemplating becoming an insider threat, the subject-wise model identified malicious intentions with 78.57% accuracy. The model also distinguished insider threat scenarios from everyday conflict scenarios with 93.47% accuracy. These findings could be utilized to support the development of insider threat mitigation systems along with existing trustworthiness assessments in the nuclear industry.

## 1. Introduction

Nuclear security involves the prevention and detection of and response to theft, sabotage, unauthorized access, illegal transfer and other malicious acts involving nuclear materials, other radioactive substances and their associated facilities [1]. According to the International Atomic Energy Agency (IAEA) Incident and Trafficking Database (ITDB), there have been 2477 confirmed incidents, 424 counts of unauthorized possession, 664 counts of theft or loss, 1337 other unauthorized activities or event, as well as the unauthorized disposal, shipment, or discovery of uncontrolled radioactive sources [2]. In particular, great attention has been paid to insider threats in nuclear facilities. As insiders have access to and authority in the facility, they have more opportunities to choose vulnerable targets and more time for malicious behavior than outsiders do [3]. Most of the known incidents of nuclear material theft and sabotage at nuclear facilities were carried out by insiders or at least with the help of insiders. Bunn and Sagan (2014) claimed that insider threats should be the most serious challenges that nuclear security systems face [4].

The well-known cases of insider incidents were the theft of highly enriched uranium (HEU) in Russia (1992) and sabotage on the Doel 4 nuclear power plant (NPP) in Belgium (2014). Unlike the other incidents in the 20th century, the sabotage of the Doel 4 NPP occurred despite having strict security and safeguard systems. Someone deliberately opened the emergency drain valve and drained 65,000 L of oil underground that brought the shutdown of the turbine for approximately five months, and this incident consequently resulted in severe economic damage. The sabotage was supposedly carried out by insiders who have not yet been identified [5].

Many recent studies have tried to systematically define insider threats at nuclear facilities, thereby suggesting ways to prevent nuclear security incidents perpetrated by insiders. Studies have focused on the systematic evaluation of physical protection systems considering the characteristics of insiders [6,7], building a security culture among members [8,9], or by developing case studies or scenario analyses of insider threat incidents [5,10,11]. Efforts have contributed to improving the security system of NPPs to be more resistant to various insider threat situations. However, these methods and regulations aim at limiting the insider’s attempts rather than preventing the threat. The prevention of an insider threat requires the detection of an insider. Such detection should include measuring and assessing potential insiders’ motivations.

In cybersecurity, effort has been made to identify and mitigate insider threats [12]. The attempts of insider threat detection have focused on investigating behavior anomalies. Hunker and Probst (2011) tried to detect insider threats in cybersecurity by monitoring insiders’ behaviors when using network and organization resources [13]. In NPPs, workers are periodically monitored through surveillance and interviewed by their coworkers and supervisors about their abnormal behavior. The workers should pass a psychological screening and drug and alcohol test to assess their fitness-for-duty. Their past records are also reviewed during a background check. Thus, the trustworthiness assessment process in NPPs is the key part of the effort to screen individuals’ potential threatening acts before and during employment.

On the other hand, the current trustworthiness assessment process needs to be improved, especially because the assessments are subjective, potentially biased, and infrequently administered [14,15]. Secondary investigation with insiders’ private information is not a robust approach to detect malintent for access [16]. Often, the assessments failed to catch indicators of potential insider problems [4]. To measure the motivations of potential insiders more reliably, the insights from previous studies should be carefully applied to the field of insider threat at NPPs.

In this regard, this study aims to examine subjects’ malicious intentions based on electroencephalography (EEG) data. We hypothesized that an EEG-based trustworthiness system, combined with self-reports, could provide objective and empirical evidence about one’s cognitive responses to committing harmful behavior. Additionally, we also examined the application of machine-learning-based algorithms to accurately distinguish the malicious intentions of subjects. To support the development of methodology to detect an insider, experimental scenarios were designed with the consideration of an NPP-related insider. Detection of an insider threat was made in comparison with detecting everyday conflict. Potential implementation issues of the proposed approach and an algorithm-driven analysis of the brain activity data of insiders were also examined considering worker practices in NPPs.

## 2. Related Work

### 2.1. Effort in Management, Regulation, and Policy in Nuclear Security for the Prevention of an Insider Threat

Since the September 11 terrorist attacks, preventive and protective measures and requirements have been introduced to strengthen physical protection systems (PPSs) and the trustworthiness of workers. Kim et al. (2017) and Zou et al. (2018) proposed a new methodology to evaluate the PPS method by calculating the detection time and probability of system failure and considering the characteristics of insiders [6,7]. In NPPs, vital areas and security measures have been reinforced assuming that the insider has enough knowledge and information. These efforts have strengthened NPP security systems.

Considerable effort has also been made to assess and screen workers’ trustworthiness and reliability. The United States Nuclear Regulatory Commission (USNRC) has extended the scope of background checks and behavior observation programs [17]. The background check includes the assessment of criminal records, references, past work history, financial records, and medical records before and during employment. In addition, psychological examinations are regularly conducted to screen potential insiders. Behavioral observation programs include the annual review and report of workers’ aberrant behaviors by their immediate supervisor [18]. Similarly, the behavior of workers is regularly observed through video surveillance inside protected areas.

However, case studies indicate that background checks and behavior observation have fundamental limitations due to their subjectivity. The attempts to categorize personalities to predict crime have achieved only limited success [19]. In fact, more effort is necessary to monitor and report suspicious behaviors of coworkers in a workplace. In the cases of Leonid Smirnov’s HEU theft in 1992 and Rodney Wilkinson’s limpet-mine sabotage in 1982, their suspicious onsite behaviors went unreported. People did not want to believe their friends and colleagues could betray the organization [20]. Especially in East Asian cultures, people rarely doubt or accuse their coworkers or superiors because individuals have been educated to respect and follow tradition and the social hierarchy [21]. This phenomenon indicates the need for using more objective indicators to overcome the limitations of the current method of trustworthiness assessment of potential insiders.

### 2.2. Detecting Insider Threats Using Biosignals

Ideally, if an insider’s malicious intention can be captured and identified in advance of taking harmful actions, the insider threat can be prevented. Some recent studies have tried to predict insider threats for such purposes by using cognitive indicators based on biosignals. For example, Suh and Yim (2018) used EEG signals to identify insider threats based on observing signature indicators while a potential insider threat is contemplating a malicious act [15]. Subjects participating in an experimental study were asked to read questions simulating situations of being forced into becoming an insider by threat or for financial gain and were asked to decide whether or not to cooperate. The study discovered a statistically significant difference in the band-to-band brain wave power ratio when choosing “yes” compared with “no” to the demand for an insider action. Specifically, the ratio of beta to alpha wavelengths as well as that of gamma to alpha wavelengths increased when the subjects were willing to perform the suggested task. In the area of cybersecurity, Almehmadi and El-Khatib (2015) proposed intent-based access control (IBAC) to reduce insider threats using the Concealed Information Test (CIT) [16]. Subjects’ biosignals were measured while they committed a mock crime. During the test session, three types of stimuli—a “probe” item, an “irrelevant” item, a “target” item—were repeatedly shown. When the biosignal was different between exposure to the “probe” and “irrelevant” items, the subject is thought to have a knowledge of the “probe” item. The study employed several types of biosignals: (1) the P300 peak of the EEG signal [16], (2) the electrocardiogram, galvanic skin response, and skin temperature [22], and (3) the number of head micromovements [23]. Hashem et al. (2018) measured the EEG and eye-tracking data while the subjects performed the mock crime [24]. Differences between simple work and unauthorized remote access activities were identified by using the time domain and frequency domain of EEG signals, eye movements and pupil sizes. Based on the subject-wise classification through the k-nearest neighbor (kNN), support vector machine (SVM), random forest, and bagging algorithms, they reported an accuracy of 89–91% using the EEG data and 69–78% using the eye-tracking data.

Previous studies have shown the possibilities as well as limitations of detecting insiders using biosignals. The scenario-reading-based approach proposed by Suh and Yim (2018) suggested using the frequency domain features of EEG signals to identify whether a subject has a malicious intention or not [15]. While the study made an important contribution by identifying potential signatures for such intention detection, the issue of intersubject variability remains to be further investigated [25].

The CIT is the most practical measure because it has been used for detecting lies for over 30 years. It could be used for access control measures in NPPs. However, preparing and presenting proper stimuli is the main limitation of the CIT. In fact, it is difficult to specify objects or scenes that are not related to people without malicious intentions and that are related to insiders with malicious intentions. In particular, an NPP is a special place with strict access control and restrictions on the use of technologies, which means that the stimuli cannot be shown to some subjects under normal circumstances.

The mock crime approach allows us to measure biosignals for an extended time period (5–30 min) with a detection accuracy greater than 90%. Compared to scenario-reading-based tasks, this approach is easier for the subjects because they can be immersed in the experiments. However, the mock crime approach has a low signal-to-noise ratio (SNR) because much movement can occur during the scenarios. Additionally, the EEG signal could reflect the characteristics of motor movement instead of complex cognitive processes. It is also difficult to obtain information about whether the subject actually has a malicious intention. In that case, using the results from these experiments may involve a high false-alarm rate.

This study further investigated the feasibility of detecting the presence of malicious intentions to identify an insider by using EEG features. The scenario reading-based approach was adopted for task design. To address the issue of intersubject variability, subject-wise classification was employed. A classification model was constructed for each subject. A subject-wise analysis has various advantages. It can overcome the moral questions related to cross-subject modeling. The feasibility and predictability of the model can be increased by continuously updating the baseline data. It is not necessary to verify the generality of the EEG signal characteristics among various personal characteristics.

### 2.3. Intention Detection Based on the Classification of EEG Signals

Several studies classified the implicit intention of subjects by employing machine learning algorithms. In a study conducted in Korea, Dong et al. (2016) asked the subjects to read the sentence presented on the monitor and then to say whether they agreed or disagreed with the content [26]. A Korean translation of the Minnesota Multiphasic Personality Inventory-2 (MMPI-2) was used [27]. The study attempted to identify the implicit intention of the subjects by including five EEG frequency domain features, such as theta, alpha, beta 1, beta 2, and gamma waves. They achieved a classification accuracy of 62–75% through SVM. The authors emphasized that this accuracy was significant because it was a single-trial experiment without multiple repetitions. From the use of functional magnetic resonance imaging (fMRI), the implicit intention in the study was found to be mostly related to the frontal lobe. The study argued that if feature extraction and classification techniques were further refined, attaining a higher accuracy would be possible.

Distinguishing different types of intentions by using biosignals was investigated in a study by Kang et al. (2015) [28]. The authors classified viewing a picture with a simple navigational purpose and searching for an object within the picture with the intention of searching for information. They compared the difference in biosignals when a subject views an identical picture with two different purposes. For the analysis of the data, two major features were taken into consideration: the n pairs with the largest potential difference among the phase-locking values and the potential differences among all the electrode pair combinations. The modeling based on SVM, naïve Bayes (NB), and Gaussian mixture model (GMM) algorithms achieved classification accuracies of 51–73%. Although the study demonstrated the utility of machine-learning-based classification of stimuli with only subtle differences, the performance was relatively low.

Classification of movement intention by using EEG signals was also examined. Bulea et al. (2014) investigated the ability to decode movement intention from EEG delta band (0.1–4 Hz) recorded immediately before movement execution [29]. The authors achieved a classification accuracy of 70–80% by using GMM based on data from epochs starting 1.5 s before a subject’s movement. Zhang et al. (2017) introduced the use of convolutional recurrent neural network models for precisely identifying human intended movements by learning compositional spatio-temporal representations of EEG signals [30]. These models showed good results with a classification accuracy higher than 90%. Similarly, Kim et al. (2019) performed reaching tasks and classified movement intention of left vs. right trials and top vs. bottom trials using EEG signals [31]. The authors achieved a classification accuracy of 73–85% through SVM.

As shown in previous studies, the machine-learning-based classification in handling EEG data has the advantage. It allows us to include a large number of metrics in a single analysis. For example, some machine-learning-based classifications used various subsets of available features and applied automatic feature selection algorithms to explore the complex brain responses during cognitive process [32]. Additionally, many deep learning models with various feature sets have been investigated for EEG classification. Deng et al. (2018) proposed the enhanced transductive transfer learning Takagi–Sugeno–Kang fuzzy system construction method to recognize epileptic EEG signals [33]. Shah et al. (2020) applied embedding reconstruction in order to design a new deep neural network. This deep neural network called Dynamical system Generated Hybrid Network for Parkinson’s disease is based on EEG signals [34]. Acharya et al. (2020) introduced a long short-term memory deep neural network to recognize four classes of negative emotions based on using frequency domain features from EEG signals [35].

This study attempts to predict implicitly expressed intention and task type at the same time by using a machine-learning-based classification based on a single experiment. Subject-wise classification was adopted to be supported by the machine-learning-driven analysis to predict the outcome with an accuracy at a satisfactory level.

## 3. Objectives and Task Design

The objective of this study was to detect insider threats based on identifying the malicious intention of a person using subjects’ brain responses in the context of nuclear facility operations. We hypothesized that brain waves show different patterns depending on the subject’s intention. The difference could be utilized to examine whether a person will become a potential insider threat.

To measure the differences in brain responses with respect to having different intentions, the subjects were assumed to be in a particular situation related to being an insider. According to planned behavior theory and the theory of reasoned action, a positive assessment of the proposed behavior increases the likelihood of taking an action [36,37]. Additionally, the clearer the intention is, the more likely the person is to take an action. Building on these findings, we provided the subjects with text paragraphs shown on a 24-inch monitor describing insider threat situations. Then, the subjects were asked whether they would agree or disagree with what was demanded in the situation. An affirmative answer, in the given situations, represents the intention to become an insider threat. As the subjects were aware of the fact that their affirmative answers do not result in committing an actual crime, they were encouraged to be candid in selecting their answers. Depending upon the type of answers chosen, the corresponding EEG signals of the subjects were observed and analyzed to identify the patterns between the intention and the responses of the signals. Accordingly, the first hypothesis formulated in this research is as follows.

**Hypothesis** **1.** *EEG signals are distinguishable depending upon whether a subject has or does not have an intention to become an insider threat.*

Suh and Yim (2018) demonstrated that a decrease in the alpha wave accompanied by an increase in the beta wave of a subject is an indication of an insider threat [15]. This result implied that a subject felt less relaxed or more alert while he or she was contemplating becoming an insider threat. However, it has not yet been identified whether such observations can be associated with the effect of everyday conflict, as facing insider threat situations is expected to increase the level of stress. It will also be of interest to examine whether the biosignals can be used to distinguish the stresses related to becoming an insider threat from the stresses of everyday conflict. Accordingly, the second hypothesis examined is as follows.

**Hypothesis** **2.** *EEG signals are distinguishable between a subject responding to insider threat situations and experiencing everyday conflict situations.*

To examine Hypothesis 1, ten different insider threat scenarios were developed. Each scenario was based on a real case referring to specific security-related incidents in NPPs. Real case incidents were used to raise the level of psychological involvement of the subjects with insider situations in an NPP during the experiments. Additionally, different levels or categories of insider threats, proposed by the IAEA (2008), were combined in the scenarios to cover diverse aspects of insider threats while minimizing redundancy.

As summarized in Table 1, ten different mixed insider scenarios were used in the study depending on the type of threat (theft or sabotage), insider action (active (violent or nonviolent) or passive), opportunity (plant operator, maintenance crew, janitorial staff, security guard, or spent fuel truck driver), and motivation (monetary gain, disgruntlement, ideology, or to bend the rules).

The types of each insider-related category (threat, action, opportunity, motivation) were randomly matched while keeping each scenario unique. In addition, technical terms related to the NPP were intentionally excluded considering that the subjects may not be familiar with NPP-specific terms. For instance, scenario #9, profiled as “sabotage – active (nonviolent) – security guard – money”, is composed as follows:

“*You are working as a security officer at a nuclear power plant in South Africa. Your role is to take care of the security of the power plant. You have worked as a nonregular worker for about a year and a half and then retired. Since then, you worked as an activist for anti-nuclear groups.*

However, to earn money, you have been recently hired again as a security guard for a nuclear power plant. Additionally, you visit your girlfriend in Zimbabwe every month. One day, your girlfriend introduces to you terrorist group members in Zimbabwe. They propose a grand action against the nuclear power plant you work for. They ask you to install a timed bomb in the reactor and control room to destroy the power plant while minimizing human casualties. After installing the bomb, you can escape. You are a security guard, and you are not subjected to a body search. There is enough time to run away after setting up the bomb.

*They offered you 5 billion won for successfully carrying out the mission. If you are arrested, you must spend five years in jail.*”

The subjects read each scenario for 30 s. Then, a question was asked: “Would you like to perform the proposed action?” the subjects replied “yes” or “no” by pressing one of two keyboards within 5 s. The data from the first 30 s period were taken for the EEG analysis to examine the intention before each subject reached their decision. The EEG signals from the 5 s decision period were not used because the signals may contain artifacts related to motor imagery for the keyboard input.

To test Hypothesis 2, another ten scenarios mimicking everyday conflict situations were developed. These scenarios were from the Korean version of the daily stress inventory (K-DSI), which describes everyday conflict situations [38]. Among the everyday conflict situations listed, those with relevance to NPP workers were selected. The scenarios included work stress, stress from work-related relationships, stress from family problems, stress from health issues, and stress from financial concerns.

Both insider threat scenarios and everyday conflict scenarios comprised three parts, i.e., introduction, main contents, and compensation for success with warning. The scenarios consisted of 82.15 (SD = 12.21) Korean words on average, and the subjects were equally exposed to each scenario for 30 s. EEG signals were recorded while the subjects were reading the scenarios.

## 4. Methods

### 4.1. Subjects

Twenty-five healthy young adults (19 men and 6 women; mean ± SD age of 25.70 ± 3.45 years) participated in the experiment. All the subjects were university students with engineering backgrounds and were voluntarily recruited. None of them had a history of psychiatric/neurological disorders or alcohol/drug dependence. The subjects were instructed to maintain a regular sleep–wake schedule with more than 6 h of sleep and not to drink caffeine or alcohol for at least 8 h prior to the experiment.

When the subjects arrived at the laboratory, they were given explanations about the experimental procedures according to the guidelines. The experiment was approved by the ethics board of Korea Advanced Institute of Science and Technology (KAIST) Institutional Review Board.

### 4.2. Experimental Protocol

The experiment room was kept dark and silent to support the subject’s concentration and to minimize the background light and sound noise. The stimuli were presented using Neurobehavioral Systems Presentation software on a 24-inch liquid crystal display monitor. The subjects were seated 50 cm away from the monitor and were instructed to wear a cap with the electrodes for EEG measurement. Additionally, the experimenter asked each subject to avoid blinking, if possible, when reading the scenarios. In addition, the experimenter explained that their answers were going to be validated through a special polygraph test. Regardless of their answers, all the subjects were given payment of the same amount at the end of the experiment. The subjects were exposed to all 20 scenarios in random order, including ten insider threat scenarios and ten everyday conflict scenarios.

### 4.3. EEG Recording and Preprocessing

**Figure 1:**
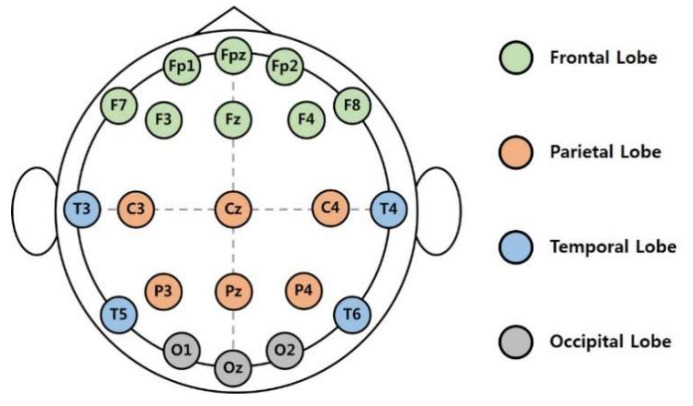
The 10–20 international system of electrode placement.

Each subject’s EEG signals were recorded using a Neuron-spectrum 4/P (Neurosoft Ltd., Russia). Each subject was fitted with an Ag/AgCl electrode cap arranged with an extended international 10–20 system. The EEG data were recorded from 21 channels (O1, O2, Oz, P3, P4, Pz, C3, C4, Cz, T3, T4, T5, T6, F3, F4, F7, F8, Fz, FP1, FP2, and FPz) at a sampling rate of 500 Hz (see Figure 1). Reference electrodes were located at both the earlobes and ground (between Fz and FPz). During the experiment, the electrode impedances of all the channels were kept below 5 kΩ. To calibrate the signals, the EEG signals were measured for 2 min with the eyes closed and 2 min with the eyes open.

To remove artifacts in the collected data, data preprocessing was performed based on Makoto’s preprocessing pipeline using EEGLAB [39]. The data were downsampled to 250 Hz, and the frequency below 1 Hz was filtered by using a high-pass filter. Then, the line noise was removed by the CleanLine plugin [40]. Bad channels were rejected using the Clean Rawdata plugin, and continuous data were corrected using artifact subspace reconstruction (ASR). All the removed channels were interpolated, and the data were re-referenced to the average potential of the 21 channels. The adaptive mixture independent component analysis (AMICA) program and postAmicaUtility toolbox were used for independent component analysis (ICA) [41]. The artifacts from moving the body, rolling eyeballs, and blinking were excluded from the analysis based on the visual inspections of each component.

The preprocessed data were divided into 2 s epochs, with a 1 s moving average for each question. Epochs smaller than 1 s were removed from further analysis to avoid duplication of the data. Each question contained up to 29 epochs, which yielded a total of 14,128 2 s epochs per channel for later analysis.

To support machine-learning-based algorithm development, a set of features that describe the EEG responses of the subjects were selected and used. We tried to explore the best use of the available EEG features in various domains. They can be categorized into four different types: time domain features, frequency domain features, time–frequency domain features, and nonlinear dynamic system features [42]. The details of these features are presented in Table 2. The features were calculated for each channel. All the features were normalized to a zero mean and unit variance across the subjects and trials.

Nine kinds of features were extracted from the time domain. Mean is the arithmetic mean of the time series. Peak-to-peak value is the difference between the maximum value and the minimum value of the time series. Skewness is an estimated value of the asymmetry of the time series. Kurtosis is an estimated value of the tailedness of the time series. Three Hjorth parameters that reflect characteristics of activity, mobility, and complexity were also extracted based on previous work [43,44].

Time–frequency domain features were calculated based on discrete wavelet transform (DWT) decomposition. DWT decomposition includes successive high- and low-pass filtering of a time series with a downsampling rate of 2. It is a suitable tool for analyzing nonstationary signals such as EEG signals because the wavelet transform has the advantages of time–frequency localization, multirate filtering, and scale-space analysis [45]. In this study, approximation coefficients (Ai) and detailed coefficients (Di) were applied using the Daubechies 4 wavelet [46]. Six coefficients were used as subbands for calculating features: D1 (62.5–125 Hz), D2 (31.2–62.4 Hz), D3 (15.6–31.2 Hz), D4 (8.8–15.6 Hz), D5 (4.4–8.8 Hz), and A5 (1–4.4 Hz). Moreover, four kinds of features were extracted for each subband. Relative energy is the wavelet energy (squared sum of the coefficients) over the total energy. Shannon entropy is the entropy in the wavelet domain, which indicates signal variations at each frequency scale. Maximum energy is the maximum value among the squared values of the coefficients. Variance is the variance in the squared sum of the coefficients.

Frequency domain features were calculated using a discrete Fourier transform (DFT). The DFT algorithm transforms the spatial domain of the analog EEG wave to the time domain, yielding spectral displays, making it easier to view the average frequency curve of the EEG record. The transformed data were categorized into seven frequency bands: delta (1–4 Hz), theta (4–8 Hz), alpha (8–13 Hz), beta (13–25 Hz), high beta (25–30 Hz), gamma (30–40 Hz), and high gamma (40–50 Hz). In addition, four kinds of features were extracted for each frequency band. Absolute power is the sum of the squared values. Relative power is the absolute power of each frequency band divided by the sum of the absolute power over all the frequency bands. Maximum power is the maximum power value. Peak frequency is the frequency that has the maximum power value.

Four features of the nonlinear dynamical system were extracted. Nonlinear dynamics and chaos theory have been applied in neurophysiology to analyze EEG signals. Nonlinear approaches have been used to discover findings that cannot be identified by conventional linear approaches [47]. Mathematical algorithms such as the approximate entropy and sample entropy were created to measure the repeatability or predictability within a time series. The correlation dimension determines the number of dimensions (independent variables) that can describe the dynamics of the system and reflects the complexity of the process and the distribution of system states in the phase space [44].

Consequently, when a subject was reading one scenario presented on the monitor, 65 features were quantified from each of the 21 channels.

## 5. Results

Among the 500 questions presented to the 25 participants, 10 questions were excluded from further analysis because the response times exceeded the limit. Therefore, 490 questions were used for further analysis. Among these questions, the answer “yes” was 246. In detail, 96 answers from the insider threat scenarios and 150 answers from the everyday conflict scenarios were “yes”. The lower number of “yes” answers for the insider threat scenarios may be due to the criminal nature of the activities involved. Table 3 summarizes the frequencies of answers from the experiment.

The collected data became the basis for the development of the EEG feature-based subject-wise classification model. To validate and test the accuracy of the classification model, we used a 1-out-of-n question cross-validation strategy. We avoided dividing the epochs for the same question into training and testing data to prevent overfitting. From the use of the 10 insider threat-related questions, the epochs from nine questions were used as the training data, and the epochs from one question were used as the testing data for the purpose of predicting answers for the insider threat scenarios. In addition, among all 20 questions, 19 questions were used as the training data, and one question was used as the testing data to classify whether the subject read the insider threat scenario or the everyday conflict scenario. The validation step was repeated until all questions were used once, as the testing data.

We used the recursive feature elimination technique to extract the most informative EEG features from the available candidate features. Variable Selection Using Random Forests (varSelRF) and the Boruta algorithms were used with their default parameters [48]. These algorithms were applied to the training data set only and the classification was limited to automatically selected features. The varSelRF algorithm uses both backwards variable elimination and highly correlated variable selection. The Boruta algorithm is a wrapper built around the random forest classification algorithm and tries to capture important features in the dataset with respect to an outcome variable. Classification was performed using various supervised machine learning algorithms to compare the effectiveness of the algorithms for distinguishing malicious intentions. For this purpose, kNN, SVM with radial kernel, NB and multilayer perceptron (MLP) algorithms were selected [49]. The parameters of the classification algorithms were tuned with the training data for each subject and for each validation step.

The classification accuracy was calculated based on whether the model correctly predicted an answer for the subject. Thus, multiple epochs in a single question should be merged into a single value because the data have a maximum of 29 epochs per question. We assumed that the predicted value of the question was “yes” if the number of epochs classified as “yes” was greater than that of “no” in a single question. Based on this assumption, we calculated the average classification accuracy for each of the feature selection and classification algorithms.

**Figure 2:**
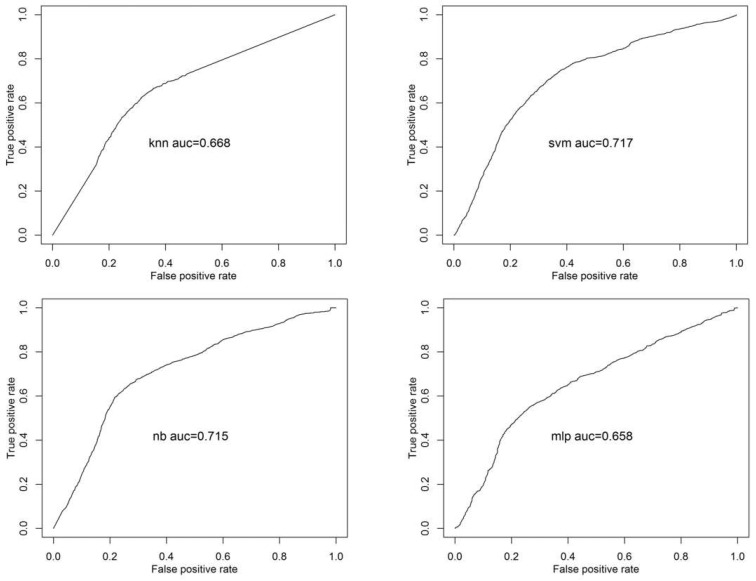
Receiver operating characteristic (ROC) curves for malicious intention detection based on using k-nearest neighbor (kNN), support vector machine (SVM), naïve Bayes (NB) and multilayer perceptron (MLP) classifiers with the Variable Selection Using Random Forests (varSelRF) algorithm.

To test Hypothesis 1, we classified the answers from the insider threat scenario data and calculated the average classification accuracy. Figure 2 shows receiver operating characteristic (ROC) for malicious intention detection. The ROC curves present classification accuracies of 65.8–71.7%. Table 4 summarizes the average classification accuracy for malicious intention detection from the feature selection and classification algorithms. The top ten important features were hjorth_mobility, hjorth_activity, sample entropy, hjorth_complexity, max (D2 wavelet), approximate entropy, permutation entropy, max (high beta), relative power of beta, and the median (see Table 2). The combination of the NB and Boruta algorithms outperformed the other classifiers with over 78% average classification accuracy, while the others achieved an accuracy of 71–74%. Two automatic feature selection algorithms showed similar overall performance, although the performance varied depending on the classifier. This result indicates that when there are baseline data for any of the nine trials, the implicit intention for a new trial can be distinguished at approximately 75% accuracy.

This work is similar to that of Dong et al. (2016) in predicting agreeing/disagreeing intentions of the subjects for a given scenario without repeatedly presenting the same scenario [50]. However, compared to the questions used in their study, more complex decision-making processes were involved in answering the questions in our study. We assumed that different kinds of complex decision-making processes could be distinguished by the classification algorithms using EEG signals. This method also requires the classification accuracy to be higher than that achieved in the previous study.

The average classification accuracy of 73–77% achieved in this study shows that the classifiers could distinguish the presence of malicious intent while considering the insider threat situation involved. In other words, the EEG signals showed different characteristics depending upon whether the subjects decided to act on the insider threat scenarios or not. Therefore, null Hypothesis 1 is rejected.

**Figure 3:**
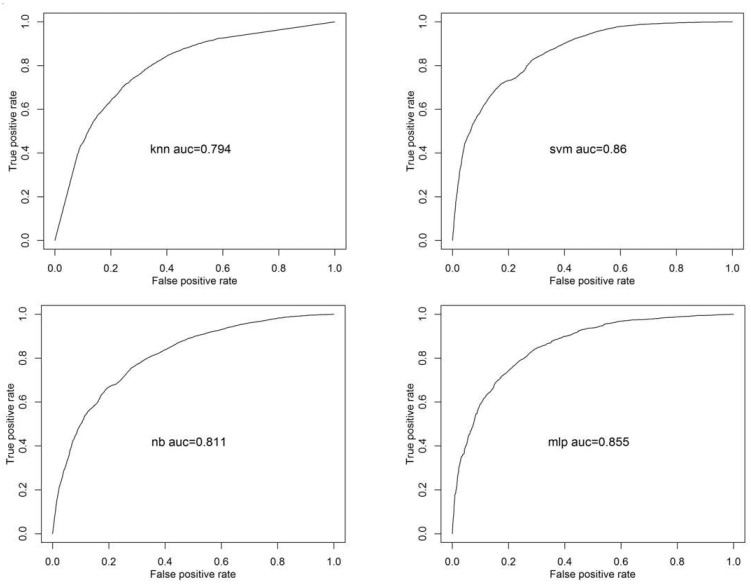
ROC curves for scenario-type detection based on using kNN, SVM, NB, and MLP classifiers with varSelRF algorithm.

To test Hypothesis 2, we classified the types of situations and calculated the average classification accuracy. Figure 3 shows ROC for scenario-type detection. The ROC curves present classification accuracies of 79.4–86.0%. Table 5 summarizes the average classification accuracy for scenario-type detection from our feature selection and classification algorithms. The top ten important features were hjorth_activity, hjorth_mobility, sample entropy, approximate entropy, hjorth_complexity, permutation entropy, max (D3 wavelet), and absolute power of high gamma, alpha, and high beta (see Table 2). The SVM algorithm with a radial kernel and the MLP algorithm outperformed the other classifiers with an approximately 93% average classification accuracy. The varSelRF and Boruta algorithms achieved similar performances.

This classification of different types of scenarios is similar to what Hashem et al. (2017) did because they distinguished the types of scenario that the subjects faced [51]. They tried to classify different kinds of tasks, while we tried to classify two kinds of scenarios with similar structures and lengths. Additionally, the amount of data and validation strategy were different. The previous study used 10 min of data per task and separated the first 7 min of the experiment into training data and the next 3 min into testing data. In this study, we used 30 s data and separated the questions as the training and testing data. Thus, we assume that the algorithms distinguish the different kinds of complex cognitive processes with EEG signals if the classification accuracy is higher than 89–91% from the study of Hashem et al. (2017).

We achieved a classification average accuracy of 90–93%, which shows that the classifiers distinguish the type of scenarios while reading the proposed insider threat and everyday conflict situations. In other words, the EEG signals responding to insider threat situations are distinctive when compared to everyday conflict situations. Therefore, null Hypothesis 2 is rejected.

We selected several subsets of channels and calculated the average classification accuracy to test the possibility of NPP application. In particular, Brodmann area 10 or the frontopolar prefrontal cortex is known to be involved in human executive function. These areas are known to be related to decision-making, empathic judgment, and self-descriptive trait judgment [52]. In a previous fMRI study involving a similar task, the superior frontal gyrus and anterior cingulate cortex were the most activated regions [50]. When considering workers’ safety, for helmets integrated with EEG electrodes, the number of attachments should be minimized while maintaining the classification accuracy as much as possible. For this purpose, we propose that the frontal lobe is the proper location for electrode attachment. We performed additional computations using the FP1, FPz, and FP2 channels corresponding to Brodmann area 10 (superior frontal cortex) and the F3, Fz, and F4 channels corresponding to the middle frontal gyrus [53]. The results are shown in Table 6.

With only three channels of data, a classification accuracy of 66–72% was achieved for malicious intention detection. In the detection of malicious intention, channels corresponding to the middle frontal gyrus achieved an approximately 1% higher accuracy compared to the channels corresponding to Brodmann area 10. This accuracy is approximately 5% lower than the accuracy using all 21 channels. When classifying the type of scenarios using three channels, an accuracy of 76–79% was achieved. This accuracy is approximately 13% lower than the results from the 21-channel data. Dong and Lee (2012) found that frontal lobes precede the conscious decision for how to answer. These findings help researchers to predict how people are going to answer in a real decision-making situation [54]. Similar to their findings, our results showed that the frontal lobes can be used to detect malicious intentions with relatively little reduction in accuracy.

## 6. Discussion

The main goal of this study was to determine whether information from EEG signals enables the detection of malicious intention, which helps to mitigate insider threats at NPPs. As we developed a subject-wise model, the model of individual EEG signals predicts the outcome of a single-trial. Therefore, the model will update its estimation when a new trial occurs. It is possible to distinguish the subject’s implicit intention by approximately 30 s through 9 or 19 scenario-based situations. A higher accuracy is expected in practice because the amount of baseline data will be larger than that from the laboratory environment. EEG signals can be obtained during various tasks throughout the day. In practice, the proposed model can provide an early warning before the insider puts an idea or plan into action. In addition, it distinguishes the two types of situations (insider threats and everyday conflict), implying that false alarms can be controlled. Some workers may continue to signal signs of everyday conflict or irritation or behave suspiciously. In this study, we tried to provide objective evidence that EEG signal-based classification contributes to the prevention of workers’ malicious intention in advance. However, we address the limitations and challenges of the study as follows.

### 6.1. Task Design

The number and length of the scenarios were limited due to concern about the concentration of the participants. In addition, similar scenarios were presented repeatedly to maintain immersion and to avoid confusion. In a real facility, the baseline signal can be measured during day-to-day work and can then be compared to the signal while performing a particular task. Especially if a real helmet is manufactured using dry electrodes, the role-playing limitation of the insider threat scenario can be overcome in a real situation. Therefore, it is possible to overcome the limitation of sentence reading and create an environment more similar to that in the real world. In this case, augmented reality or virtual reality technologies can be used to interact with others in a virtual environment. In addition, robustness to countermeasures has not been verified. In this study, applying lie detectors helped subjects have very little motivation to lie.

### 6.2. Feature Engineering and Modeling

To detect implicit intention for insider threats with the highest possible accuracy using EEG signals, it is necessary to use all the available features. However, this method may result in overfitting due to a large number of features. In fact, we followed an automatic feature selection process and strictly differentiated the training and testing sets. Instead, the classifiers used in this study reported relatively similar classification accuracies. This finding provides evidence to overcome the overfitting problem. In future work, the model could be further optimized using different epoch lengths and additional features.

### 6.3. Application to Nuclear Facilities and Implications for Nuclear Security Culture

The ideal way to use biosignals to identify whether a person is involved in an insider threat is to catch an insider’s malicious intention and trigger an alarm before an action is taken. However, biosignal studies to date cannot provide a means to predict who will actually commit a crime. If the crime has already been committed to some extent, there is a possibility of measuring the difference in psychological and cognitive responses.

In case studies of insider threats, people around the insider had observed the indicators of malicious intentions or suspicious behavior without showing any proper responses. Generally, insider threats are secretly planned over long periods of time, from months to years. Therefore, even if it is difficult to identify an insider’s criminal intentions without any error, the methodology proposed in this study can be used as a supplementary tool to assess the trustworthiness of workers and to identify potential insider threats. It could help develop criteria for excluding the potential insider threat temporally from the workplace, which would enable the efficient use of limited security resources and contribute to reducing insider threat motivations.

Furthermore, previous studies suggested the use of EEG electrodes attached to the inside of the helmet when harvesting brain responses [55]. This type of mobile application could accrue several benefits, such as real-time fitness-for-duty checks for workers. Studies indicating such applications include those for worker fatigue detection [44], accident and alcohol detection for fitness-for-duty checks [56], and smart avionics studies to reduce human error of pilots [57]. As wearing a helmet in NPP sites is mandatory, EEG electrodes can be integrated into the safety helmet. The collected EEG data from the safety helmet can be processed and analyzed to monitor malintent and eventually to improve human performance. The results indicate the possibility of implementing a safety helmet idea in actual NPP sites. As proposed above, malicious intention was mainly dependent on the frontal lobe, the region where electrodes can be easily attached to the safety helmet. A higher classification accuracy is expected, as far as an advanced helmet design supports the necessary sensor attachment. For future work, it is necessary to test diverse subsets of features and various parts of the brain to optimize the suggested helmet design.

A preparation period is required to commit theft in or sabotage a nuclear facility. Thus, the biosignal does not need to be analyzed in real-time to detect an insider threat. EEG data could be measured while working and stored and analyzed after work. Routine inspections can be carried out on those authorized to enter a particular area. Individual baseline EEG data could be continuously collected. The model could be used as an alarm in a specific place with personal data anonymized and without indicating any specific person.

Due to the variable nature of EEG signals, false-positive signals may occur in the application of the proposed approach. Therefore, careful examination of the positive results from the classification model is necessary. While a person may develop a malicious intent under certain circumstances, that person does not necessarily become an insider threat. While the proposed approach may be used to identify a potential insider threat, more importantly, the approach can be used to strengthen the security system of a facility or to support the development of nuclear security culture. The model proposed in this study can be used as a means to weaken insider motivation while strengthening trust and reinforcing security culture among employees.

There may be moral or legal questions about the collection and evaluation of EEG data on an individual basis. The monitoring approach yields inevitable conflicts between the security interests of the organization and the privacy interests of individuals [14]. National laws may restrict identity verification and trustworthiness assessments in a state [3]. Even after the collection of biosignals is legally allowed, there may be an issue that all workers are suspected to be potential insiders. This assumption could irritate workers and have a negative effect on the security culture. Nonetheless, there have been studies using biosignals for fitness-for-duty assessments and human error prevention [58]. If the purpose of collecting biosignals apparently helps workers work more safely and efficiently, various approaches to trustworthiness assessment will be positively considered.

## 7. Conclusions

This study verified and improved the feasibility of using EEG signals for insider identification in the context of NPPs. To classify whether the subject has a malicious intention or not when facing an insider threat situation, a subject-wise classification model was suggested based on multichannel EEG data. The classification model distinguished the existence of malicious intention while the subjects read insider threat scenarios with 78.57% accuracy from EEG signals. In addition, the model distinguished the difference in the EEG signals between the insider threat situation and the everyday conflict situation with 93.47% accuracy. The classification model could provide objective evidence to strengthen the security of a facility by effectively supplementing existing trustworthiness assessments, leading to the prevention of an insider threat.

## Figures and Tables

**Table 1:** Ten insider threat scenarios.

No.	Threat Type	Insider Action	Insider Attempt with Opportunity	Insider Attempt with Motivation
1	Theft	Passive	Plant operator	Bend the rules
2	Theft	Passive	Janitorial staff	Money
3	Theft	Passive	Maintenance crew	Ego/disgruntlement
4	Theft	Active, nonviolent	Plant operator	Ideology
5	Theft	Active, nonviolent	Security guard	Money
6	Sabotage	Passive	Security guard	Money
7	Sabotage	Passive	Maintenance crew	Ego/disgruntlement
8	Sabotage	Active, nonviolent	Truck driver	Ideology
9	Sabotage	Active, nonviolent	Security guard	Money
10	Sabotage	Active, violent	Security guard	Ego/disgruntlement

**Table 2:** Extracted electroencephalography (EEG) features in the four main categories.

Feature Type	Extracted Features
Time domain (9 features)	Mean, mean square, median, peak-to-peak value, skewness, kurtosis, Hjorth parameter: activity, mobility, and complexity.
Time–frequency domain (24 features)	Wavelet bands: detailed coefficient 1–5 and approximate coefficient 5. Four features: relative energy, Shannon entropy, maximum energy, and variance.
Frequency domain(28 features)	Frequency: delta, theta, alpha, beta, high beta, gamma, and high gamma. Four features: absolute power, relative power, maximum power, and peak frequency.
Nonlinear dynamical system (4 features)	Approximate entropy, sample entropy, permutation entropy, correlation dimension.

**Table 3:** Frequencies of answers.

Answer	Insider Threat	Everyday Conflict	Total
No	146	98	244
Yes	96	150	246
No response	8	2	10

**Table 4:** Average classification accuracy for malicious intention detection.

	kNN	SVM	NB	MLP
varSelRF	73.11%	73.95%	77.73%	73.52%
Boruta	73.94%	74.37%	78.57%	71.01%

**Table 5:** Average classification accuracy for scenario-type detection.

	kNN	SVM	NB	MLP
varSelRF	92.24%	93.27%	90.41%	93.47%
Boruta	90.82%	93.06%	91.43%	92.24%

**Table 6:** Average classification accuracy using specific brain areas with varSelRF.

	Brain Area	kNN	SVM	NB	MLP
Detection of malicious intentions	Brodmann area 10 (FP1/FPz/FP2)	66.81%	69.33%	71.01%	68.91%
	Middle frontal gyrus (F3/Fz/F4)	68.07%	68.49%	72.27%	70.59%
Detection of the type of scenarios	Brodmann area 10 (FP1/FPz/FP2)	77.35%	77.96%	78.98%	77.96%
	Middle frontal gyrus (F3/Fz/F4)	76.33%	78.98%	79.18%	76.73%
